# Engineering switchable and programmable universal CARs for CAR T therapy

**DOI:** 10.1186/s13045-019-0763-0

**Published:** 2019-07-04

**Authors:** Delong Liu, Juanjuan Zhao, Yongping Song

**Affiliations:** 1 Department of Oncology, The First affiliated Hospital of Zhengzhou University, Zhengzhou, China; 2 Department of Medicine, New York Medical College and Westchester Medical Center, Valhalla, NY USA; 3 The affiliated Cancer Hospital of Zhengzhou University and Henan Cancer Hospital, 127 Dongming Road, Zhengzhou, 450008 China

**Keywords:** Chimeric antigen receptor, CAR T, Universal CAR, scFv

## Abstract

A traditional chimeric antigen receptor (CAR) has a fixed design, and one type of CAR T cells can only target one antigen epitope. This rigid design limits clinical application and leads to exceptionally high manufacturing cost. New CARs are being engineered with a modular approach so that the antigen recognition domain is split from the signaling domain of a conventional CAR, hence the target antigen can be switched or re-directed more readily without the requirement of re-engineering the CAR T cells. This CAR system can therefore serve as a universal CAR (UniCAR). The UniCAR platform has a modular design that splits the conventional CAR to 2 separate components: (1) a signaling module that binds to a specific epitope on the switching molecule and (2) a switching module with an antigen-binding domain and a switching epitope specifically recognized by the signaling module. A variety of switchable CARs have been engineered. The switchable modular designs include the dimerizing platforms using leucine zippers and biotin-avidin system, and the neo-epitope tagging platform using FITC, 5B9, and PNE. The switch molecule serves as a synapse between the CAR T cells and the target tumor cells. The universal CAR platforms are highly versatile, are easily re-programmable, and therefore have a vast potential for broad application and may significantly lower the cost of CAR T cell therapy. However, the current modular design of the switching molecules relies on adding exogenous sequences/epitopes. These unnatural epitopes can potentially lead to new antigenicity which may lead to generation of blocking antibodies. Furthermore, the generation, preparation, and clinical applications of the switching modules per se may involve additional clinical trials and regulatory examination for safety and efficacy, since repeated administrations of these molecules/“drugs” are anticipated. Thus, these switching molecules and UniCAR CAR T cells may require separate clinical trials and invoke different regulatory processes. This whole field is medically appealing and could present new challenges in the development of novel immunotherapeutic agents.

## Introduction

Chimeric antigen receptor (CAR) T cell therapy is a targeted cellular immunotherapy that uses genetically engineered T cells to specifically eliminate the antigen-bearing tumor cells [1–7]. CAR T therapy has achieved encouraging results in both preclinical and clinical researches of a variety of tumors [7–13]. It is considered as a revolution in cancer immunotherapy and is leading to a paradigm shift in cancer management. Two such CAR T cellular products have been approved for clinical applications [14–18]. However, significant complications with cytokine release syndrome (CRS) and CAR T-related encephalopathy syndrome (CRES) are major clinical hurdles [19–22]. In addition, laborious and costly manufacturing process of the current form of autologous CAR T cells also seriously hinders the broad clinical application of CAR T therapy [23, 24]. A traditional CAR consists of an antigen-specific single-chain variable fragment (scFv) fused with an intracellular signaling domain via a hinge and transmembrane region [4–7]. The CAR engineered in this way can only recognize one specific target, and the corresponding CAR T cells can therefore only be used for one specific type of antigen-containing cancer cells. To target a different antigen epitope on even the same cancer cell, a new CAR and the CAR T cells have to be re-engineered and manufactured. Due to the fixed design of the traditional CAR, the magnitude of activation and cytokine release after CAR T cell infusion is poorly predictable and often difficult to control. This can lead to high incidences of CRS and CRES which can be lethal if not appropriately managed [19].

Currently, efforts are being made to boost the specificity, attenuate antigen escape, and improve the effectiveness of CAR T cells. To achieve this, dual and triple CARs capable of simultaneously targeting two or three antigens respectively have been designed [25–29]. Nevertheless, according to a recent clinical trial of CD22 CAR T cells for the treatment of patients relapsed after CD19 CAR T cell therapy, tumor cells were still able to evade the killing of genetically programmed T cells by losing or downregulating both antigens [30, 31]. To reduce the toxicity associated with CAR T cell therapy, suicide genes or transient mRNA CAR were applied to remove CAR T cells when necessary or shorten their lifespan [32–36]. These are considered as fourth-generation CARs. Another approach is to use a CAR that can be inhibited when bound to antigens in normal tissues [34], but it only restricts the CAR T cells in normal tissues to some extent. All these measures have focused on fine-tuning the fixed CAR designs and/or on controlling the persistence of CAR T cells.

To avoid the costly manufacturing process of engineered T cells and evade antigen escape as well as to broaden the targeting of complex tumor antigens, new CARs are being engineered with a modular approach so that the antigen recognition domain is split from the signaling domain of a conventional CAR, hence the target antigen can be switched or re-directed more readily without the requirement of re-engineering the CAR T cells. This CAR system can therefore serve as a universal CAR (UniCAR).

**Fig. 1:**
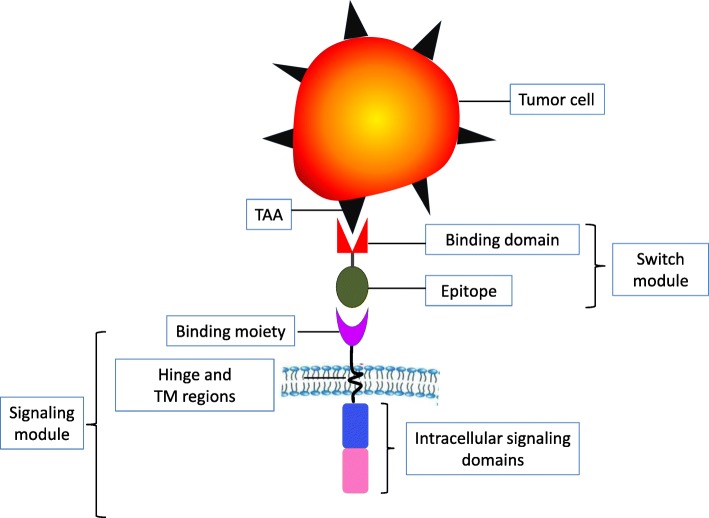
The schematic structure of a universal CAR system. The universal CAR (UniCAR) system splits the conventional CAR into two modules. The two modules are (1) the signaling module, which harbors a binding moiety to a specific epitope combined with the intracellular signaling domains via hinge and the transmembrane regions (TM) and (2) a switching module, which is a bispecific fusion molecule with one binding domain directed against a tumor-associated antigen (TAA) and an epitope specifically recognized by the signaling module

The modular design of the UniCAR platform consists of 2 separate components: (1) the signaling module, which harbors a binding moiety to a specific epitope and (2) a switching module (SM), which is a bispecific fusion molecule with one binding domain directed against a tumor-associated antigen (TAA) and an epitope specifically recognized by the signaling module (Fig. 1, UniCAR system).

**Fig. 2:**
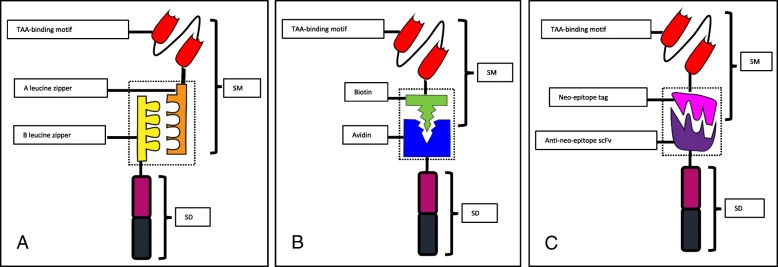
The schematic structures of switching modules. **a** SUPRA CAR. A switch molecule (SM) with a leucine zipper linked to an antigen-specific scFv (TAA-binding motif) (A leucine zipper) paired with a cognate leucine zipper-containing universal receptor (B leucine zipper) linked to the intracellular signaling domain (SD) of the engineered T cells. **b** BBIR CAR. BBIR CAR is composed of an extracellular avidin motif linked to an intracellular signaling domain. The switch molecule is a biotinylated antigen-binding motif, i.e., a monoclonal antibody, scFv, or other tumor-specific ligands that selectively bind to avidin within the BBIR. **c** Switch modules with neo-epitope tagging. In this platform, the switching modules contain a neo-epitope that is tagged to a TAA-binding motif. The CAR is composed of a neo-epitope-specific scFv linked to the intracellular SD domain. These neo-epitopes are usually exogenous amino acid epitopes that are not present in human. Three tags are currently explored, FITC, 5B9, and PNE

**Fig. 3:**
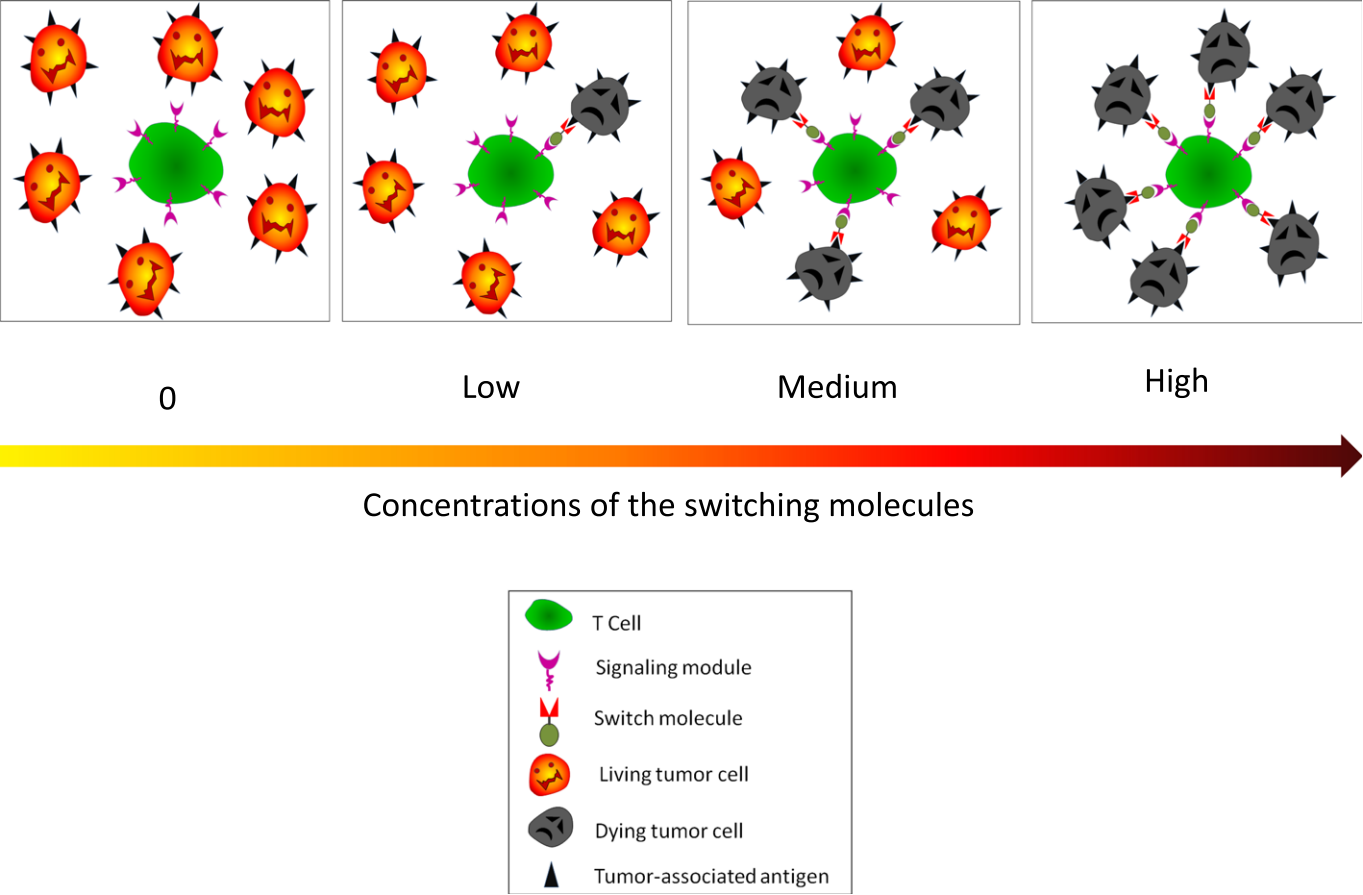
The applications and regulations of the universal CAR (UniCAR) system. UniCAR T cells cannot lyse tumor cells without a switching molecule (concentration 0) and can only kill a limited number of tumor cells at low concentrations. The killing potency of the UniCAR T cells can be adjusted and programmed by changing the concentration of the switching molecules. This system can be programmed to fine-tune the activity of the UniCAR T cells or to shut it down with the cessation of administration of the switch molecules

**Fig. 4:**
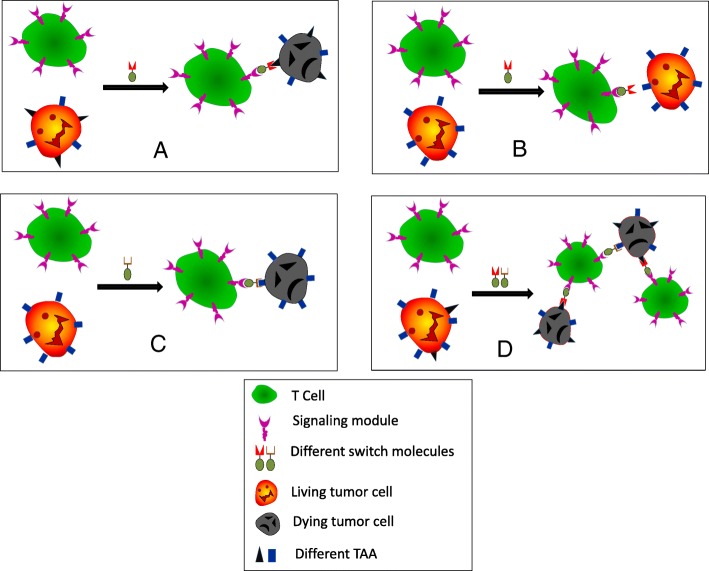
Multi-targeting of the universal CAR (UniCAR) T cells. UniCAR T cells can lyse tumor cells expressing the corresponding target antigen in the presence of the switch molecule (**a**). When the tumor cells lose the specific antigen, the UniCAR T cells could not recognize the tumors cells. This can lead to cancer resistance and relapse (**b**). By adding a second switch molecule that targets a new TAA, the killing function of the same UniCAR T cells are reactivated (**c**). Simultaneous or sequential addition of a variety of switch molecules targeting different TAAs can direct the same UniCAR T cells to more efficiently lyse tumor cells (**d**). This enhances the potential CAR T cells to combat cancer resistance and relapse

The engineered CAR T cells expressing the UniCAR remain constant and do not need re-manufacturing for each new target. The UniCAR T cells stay inert until a bispecific switch molecule crosslinks the T cells to the specific target cells. Therefore, the switch molecule serves as a synapse between the CAR T cells and the antigen-bearing tumor cells. Currently, several switching modules have been examined (Fig. 2). The extent of the response of the UniCAR T cells is strictly related to presence of the switch molecule and the corresponding antigen. The magnitude of the response can be titrated by adjusting the concentration of the SM, making it possible to switch the CAR T cells on and off and dial the CAR T cell reactivity up and down (Fig. 3, UniCAR applications and regulations). The switching molecules may therefore be considered as a “drug” and can be administered repeatedly whenever it is needed. Accordingly, side effects associated with the UniCAR T cells can be controlled by an interruption of the administration of the SM. It is also possible to use multi-specific SM molecules simultaneously or sequentially to target multiple epitopes/antigens on one type of tumor (Fig. 4, multi-targeting network). This may avoid antigen escape/antigen loss and prevent relapse or refractory diseases.

In this review, we summarized the new development in engineering the switchable and re-programmable universal CARs.

## Switch modules with a dimerizing platform

### SUPRA CAR: split, universal, and programmable

The SUPRA CAR system is a split, universal, and programmable (SUPRA) CAR with two components: a leucine zipper-containing universal receptor (zipCAR) linked to the intracellular signaling domain of the engineered T cells and a separate switch molecule with a cognate leucine zipper linked to an antigen-specific scFv (zipFv) (Fig. 2a) [37]. Once the leucine zippers pair with each other, the extracellular signal can be transmitted to the intracellular domain and the engineered T cells are activated. If a zipFv has no antigen-specific scFv, then the T cell activation is quenched. For the same mechanism, a leucine zipper with various binding affinities to the cognate zipCAR can be used to compete with a scFv-containing zipFv, thereby modulating the signal strength.

Since the scFv in the switch molecule could be discretionally changed as desired and the zipCAR T cells can remain constant, the same batch of zipCAR T cells can be used to target multiple tumor antigens simultaneously or sequentially without genetic re-engineering [37]. This property of the SUPRA CAR system thus has the potential for a vastly expanded tumor-targeting repertoire, conferring SUPRA CAR T cell system the potential to counteract antigen escape, increase effectiveness, and reduce recurrence through multi-targeting of different antigens/epitopes. The responsiveness of the SUPRA CAR T cells can be controlled and fine-tuned by adjusting different affinities between the leucine zippers and the concentrations of the added switch molecules [37, 38]. The activated SUPRA CAR T cells could also be shut down when necessary by adding a competitive zipFv that contains no antigen-binding scFv. The adjustable and programmable SUPRA CAR design therefore makes it possible to regulate the signal strength and tune down the adverse events such as CRS and CRES, thereby increasing the safety of CAR T therapy in vivo.

The SUPRA CAR system has been shown to be effective in vitro and in mouse models of leukemia and solid tumors [37]. A clinical trial of this SUPRA CAR system is needed to confirm the vast potential of the promising UniCAR design.

### BBIR CAR: biotin-binding immunoreceptor

The biotin-binding immunoreceptor (BBIR) represents another split and switchable CAR design (Fig. 2b) [39–41]. The BBIR CAR is composed of an extracellular avidin motif linked to an intracellular signaling domain. The switch molecule is a biotinylated antigen-binding motif, i.e., a monoclonal antibody, scFv, or other tumor-specific ligands that selectively bind to avidin within the BBIR [40]. Simultaneous binding of the biotinylated switch molecules and tumor cells expressing the specific antigens to the BBIR activates the BBIR CAR-engineered T cells, leading to lysis of the tumor cells [42].

The BBIR CAR T cells containing extracellular dimeric avidin (dcAv) can efficiently recognize and bind to a variety of biotinylated molecules including full-length antibodies, scFvs, and secreted cytokines such as IFN-γ [40–43]. The biotinylated antibody at as low as 0.1 ng/ml has been shown to elicit immune response of dcAv BBIR T cells and trigger cytokine secretion [40]. Even though dcAV has high affinities, supraphysiologic concentrations of soluble biotins did not cause activation of BBIR CAR T cells [40], confirming the safety of the BBIR CAR. With the split and switchable CAR design, the BBIR CAR system has the similar versatility as a universal CAR since it is switchable, adjustable, and programmable [39–41]. In vitro, the dcAv BBIR T cells showed comparable ability of lysing target cells [41] and cytokine secretion [40] to traditional CAR T cells in the presence of corresponding switching molecules. Moreover, in a human ovarian cancer mouse model, the robust tumor clearance ability of BBIR T cells was initiated by the corresponding specific switch molecules [40]. The clinical potential of the BBIR CAR T cells must be tested in clinical trials.

## Switch modules with neo-epitope tagging

### Anti-FITC CAR: FITC tagging

Fluorescein isothiocyanate (FITC) is a synthetic dye that is physiologically deficient in human body and has been used widely as a flowcytometry marker to label antibodies. Folate receptor is frequently overexpressed in solid tumor cells. Therefore, Folate-FITC hybrid molecule is used as a small molecular switch in engineering a switchable CAR [44]. The anti-FITC CAR contains two essential elements, one is a FITC-tagged tumor-targeting switch molecule [45, 46] and the other is a transmembrane CAR with an anti-FITC scFv (Fig. 3c). The FITC-labeled switch molecules form a pseudo-immunological synapse between anti-FITC CAR T cells and tumor cells expressing a given antigen, such as folate receptor or CD19, leading to lysis of the corresponding tumor cells.

In this anti-FITC scFv CAR system, the CAR T cells with the anti-FITC scFv CAR remain constant. Major efforts have been made on constructing FITC-tagged switch molecules. Two different types of switch molecules have been designed to bind to the anti-FITC-based CAR T cells. One was FITC-conjugated folate which can bind to folate receptor (FR)-overexpressing tumor cells [47–51]. In this system, an anti-FITC scFv CAR was constructed and linked with a 4-1BB costimulatory domain. In the presence of folate-FITC switch molecules, the anti-FITC CAR T cells were directed to FR-overexpressing tumor cells. It was demonstrated that the FR negative cells were not targeted, indicating that the folate-FITC switching molecule can specifically bridge and direct CAR T cells to the target tumor cells.

FITC was also conjugated to anti-CD19 and anti-CD22 scFvs to engineer CD19- and CD22-targeted switch molecules. This process involves site-specific modification of immunoglobulin proteins [45]. To allow precise control of conjugation site and stoichiometry of modified molecules, specific site-directed modifications were done via enzymatic or chemical process [52]. In recent years, recombinant methods through DNA engineering have been adopted to incorporate unnatural amino acid (UAA) residues into the target peptide. These inserted amino acid residues can serve as chemical handles for conjugation of exogeneous molecules to the peptide. These UAAs are carefully chosen so that the structure of the wild- type protein is minimally perturbed, and the UAA-modified protein is ready for further conjugation without additional manipulation. Using this method, FITC was conjugated to anti-CD19 and anti-CD22 scFvs. This site-specific protein conjugation strategy involved noncanonical amino para-azidophenylalanine (pAzF) at six exposure locations (A, G68; B, S74; C, T109; D, A121; E, S202; and F, K138). Furthermore, the crystal structure of the antigen-binding regions (proximal A and B, medial C and D, distal E and F) in the murine CD19-specific-Fab 93f3 was used to clarify the effect of FITC conjugation sites on the distance and geometry of pseudo-immunological synapse structure [45]. The FITC-labeled switch molecules were confirmed for their specific binding to the targeted antigens [45]. It was also noted that bivalent FITC conjugates (AB, EF) had stronger affinity for anti-FITC CAR T cells and led to stronger effector functions than the corresponding monovalent [45]. However, the scFvs with different affinities to FITC appeared to elicit similar degree of CAR T cell activation from the same switch molecule [53], suggesting that specificity rather than affinity to FITC plays a major role in the CAR T cell activation.

Since the same batch of anti-FITC CAR T cells could be used without re-engineering, and the switch molecules can be remodeled easily according to target antigens, the flexibility of this system makes the CAR T cells switchable and re-programmable to target multiple tumor antigens [45, 46]. Anti-FITC CAR T cells, when combined with optimally designed switch molecules (for instance anti-CD19 AB-FITC), showed tumor-specific responses comparable to traditional CAR T cells as evidenced by tumor lysis, upregulation of activation markers, cytokine secretion, and induction of costimulatory signaling [45]. The optimal dose of the switch molecules required for triggering anti-FITC CAR T cells to clear tumor cells was determined in a xenograft model [45]. This anti-tumor activity and cytokine secretion could be regulated simply by adjusting the concentrations of the switch molecules. The CAR T cell activation was shown to be interrupted by simply ceasing administration of the switch molecules [45]. Therefore, this FITC-tagged switchable and programmable CAR system offers an alternative venue for engineering universal CARs. Clearly, clinical trials are needed to confirm the therapeutic activity as well as safety.

### Anti-5B9 CAR: 5B9 tagging

5B9 is a 10-amino acid (aa) non-immunogenic peptide motif derived from La/SS-B which is an autoantigen known in Sjögren’s syndrome and systemic lupus erythematosus [54–56]. The scFv targeting the 5B9 motif has been employed to construct a UniCAR system that splits the antigen-binding domain and the signaling domain of the CAR into two separate parts (Fig. 2c). The two separate components of the anti-5B9 CAR were (a) a 5B9-binding CAR structure consisting of an anti-5B9 scFv linked by a hinge and transmembrane region to intracellular signal domains and (b) a switch molecule containing a tumor antigen-binding moiety fused to the 5B9 tag [54, 57, 58].

The 5B9-specific UniCAR system was tested in AML cells expressing both CD33 and CD123 surface molecules [57]. In this study, monospecific switching molecules against CD33 and CD123 were engineered. Furthermore, bispecific SM against both CD33 and CD123 were also made. These SMs were tagged with the 5B9 epitope. Application of two independent monospecific switch molecules simultaneously or the bispecific switch molecules simultaneously targeting the two tumor antigens resulted in effective cell lysis in vitro and supported a promising platform for multi-antigen targeting [57, 59]. Furthermore, bispecific switch molecules were shown to be more effective than simultaneous application of the monospecific switch molecules, which may be related to the competitive binding of the two independent switch molecules to the same CAR [57]. Quantitative experiments showed that switch molecules even at very low concentrations could effectively induce lysis of target tumor cells, irrespective of the densities of target antigens [57]. This study also confirmed that the UniCAR-directed T cell cytotoxicities can be titrated with different concentrations of the SM molecules, as those depicted in Fig. 3, and dual-targeting CAR T cells are more efficient than mono-targeting CAR T cells, as those depicted in Fig. 4.

The anti-5B9 UniCAR system has been characterized in a prostate cancer solid tumor model [60]. In this system, an scFv against the prostate stem cell antigen (PSCA) was tagged with the 5B9 epitope, forming a switching molecule that targets PSCA-containing carcinoma cells. A 5B9-specific scFv-containing UniCAR was engineered into T cells. This UniCAR T cell platform was shown to have potent anti-tumor potential for solid tumors such as prostate cancer in vitro and in animal models [60, 61].

Although autoantibodies against La antigen were usually present in patients with Sjögren’s syndrome [55, 56], they can be detected in people without the autoimmune disease. Therefore, these autoantibodies can interfere with the recognition between CAR and 5B9-tagged switch molecules. Fortunately, no autoantibodies against the 5B9 peptide motif applied in the switch molecule were found from large-scale screening of the sera from patients with systemic lupus erythematosus and Sjögren syndrome [62].

### Anti-PNE CAR: peptide neo-epitope tagging

A new platform of switchable CAR has been designed by using a peptide neo-epitope (PNE) in the switch molecule [63]. In this platform, the switch molecule contains a peptide of 14-aa residues. This 14-aa PNE tag can be specifically recognized by the signaling CAR containing a PNE-specific scFv (Fig. 2c). This 14-aa PNE is found in the yeast transcription factor GCN4 and was genetically inserted at the defined site of a tumor antigen targeted scFv. The 14-aa PNE of yeast GCN4 does not exist in the human body [64, 65]. The immunogenicity analysis through in silicon modeling showed that this sequence had a low possibility of evoking immunogenic response and supported the safety of the sequence in the switch molecule [63]. Thermal melting analysis further showed that implantation of the 14-aa PNE tag at the specific sites of the scFv had least effect on antigen binding and protein stability [63].

A PNE-specific switchable CAR was created by using a 52SR4 antibody scFv with high affinity for the PNE [66]. This PNE-specific scFv was fused into a second-generation CAR structure consisting of the CD8 hinge and transmembrane regions fused to the 4-1BB and CD3ζ intracellular signaling domains [63]. A CD19 specific scFv was fused to the PNE tag and was tested as a switch molecule. The PNE tag was also engrafted into both heavy and light chains of CD19-specific scFv to create a bivalent switch molecule. PNE-specific CAR T cells have been shown to bind to the PNE-tagged CD19-targeted switch molecules exclusively, while untransduced cells cannot, and the antigen targeting region of the switch molecules could redirect CAR T cells to lyse CD19+ B ALL leukemia cells [63]. The switchable CAR T cells were also shown to be active against the Nalm-6 ALL in xenograft mouse model. In this PNE-specific switchable CAR platform, site and valency as well as dose-dependent control of CAR T activity were confirmed both in vitro and in vivo. This PNE-tagged switch molecule can be easily engrafted to different scFvs and thereby expands the potential for broad application in switchable CAR engineering.

## Future perspectives

The switch molecules serve as a synapse between the CAR T cells and the target tumor cells. The magnitude of the response can be titrated by adjusting the concentration of the switch molecules, making it possible to switch the CAR T cells on and off and dial the CAR T cell reactivity up and down. The side effects associated with the CAR T cells can be controlled by an interruption of the administration of the switch molecules. The universal CAR platforms are highly versatile, easily re-programmable, and therefore have a vast potential for broad clinical applications. Since the signaling modules on the CAR remain constant and the same CAR T cells can be used to target any SM-directed tumor cells, the UniCAR system may significantly lower the cost of CAR T cell therapy. The modular UniCAR designs can be engineered into third-party “off-the-shelf” universal T cells. A universal CD19-targeted CAR T product has been tested in adult and pediatric patients with R/R ALL [67–70]. There is a potential to combine the UniCAR designs with the universal CAR T cells so that the UniCAR T cells can be used to target any tumor for virtually all patients.

However, the current modular design of the switching molecules relies on adding exogenous sequences/epitopes. This process requires complicated recombinant engineering processes and also known target sequences. Furthermore, unnatural epitopes added to these tumor antigen scFvs can potentially lead to new antigenicity which may lead to generation of blocking antibodies. The potential risks of these approaches in human subjects remain unknown. Finally, the generation, preparation, and clinical applications of the small switching modules per se may involve additional clinical trials and regulatory examination for safety and efficacy, since repeated administrations of these molecules/“drugs” are anticipated. Thus, these switching molecules and UniCAR CAR T cells may require separate clinical trials and invoke different regulatory processes. This whole field is medically appealing and could present new challenges in the development of novel immunotherapeutic agents.

## Conclusions

The modular UniCAR designs make it possible to engineer a variety of switchable and programmable UniCARs. The combination of the universal CARs with universal T cells offers virtually unlimited potential for cancer immunotherapy, though clinical trials are urgently needed to confirm the safety and efficacy of these switching molecules and switchable/programmable universal CAR T cells.

## Data Availability

The material supporting the conclusion of this review has been included within the article.

## Abbreviations

BBIR: Biotin-binding immunoreceptor
CAR: Chimeric antigen receptor
CRS: Cytokine release syndrome
FITC: Fluorescein isothiocyanate
PNE: Anti-peptide neo-epitope
scFv: Single-chain variable fragment
SUPRA CAR: Split, universal and programmable CAR
TAA: Tumor-associated antigen
